# Supplementary material for: The highly heterozygous European amphioxus (Branchiostoma lanceolatum) at the edge of panmixia
Source: PLoS Genet. 2026 Sep 8;22(9):e1012293. doi: 10.1371/journal.pgen.1012293 (PMC13577491; doi:10.1371/journal.pgen.1012293)
Supplement: Supplementary file 1 [file pgen.1012293.s016.pdf]

Response to Reviewers

Reviewer #1:

The authors provide an analysis of the genetic diversity of European amphioxus across two populations in the waters of France. They confirm the striking levels of heterozygosity that have previously been reported for various species of amphioxus and go on to provide some careful analyses that demonstrate this animal appears to exhibit panmixis, even across these distant populations. The manuscript is very clearly written and the importance of the work clearly justified. This establishes amphioxus as an extremely widespread marine species that serves as an ideal study system for understanding the evolutionary dynamics and mechanisms underpinning genetic diversity. The approach described by the authors in this study can be taken as the 'gold standard' for how genetic diversity should be measured in a species, with implications well beyond amphioxus research.

I find myself in the pleasurable (and highly unusual) position of not having any substantive questions or recommendations for revisions, since this is such a well-written manuscript and the analyses are thorough and clearly explained.

We are extremely grateful to the reviewer for this kind comment. While it is uncommon to receive such recognition from an expert in a peer review, it is vital for building a strong, harmonious scientific community that enhances collaboration and promotes high-quality research.

My only comment is whether each of the individual genome assemblies will be available in the data release? These will be extremely useful to the community, particularly given the high genetic diversity in this species, as the assemblies can be used to help locate functional and constrained genomic features. However, my reading of the data availability statement makes me think that the authors plan to only release the raw sequence reads. This would be a missed opportunity, in my view.

We thank the reviewer again for this comment. We think it is a very important point to raise and we made the VCF file with all the variants called in all the individuals of our sample available in Zenodo (https://doi.org/10.5281/zenodo.21503009).

There are also a very small number of typos, as follows.

The authors say they have analysed Ciona intestinalis, but if they have used the KH assembly then I think that they mean that they have used Ciona robusta (even though the database itself still refers to the old naming of this species as intestinalis).

Thank you for pointing this out. We have corrected the *Ciona* species name in the manuscript text and figures.

Line 411: "This is we split" does not make sense and needs amending.

Corrected, thank you.

Line 430: change “Shortly” to “Briefly”.

Changed, thank you.

Reviewer #2:

In the manuscript entitled "The highly heterozygous European amphioxus (Branchiostoma lanceolatum) at the edge of panmixia", the authors present a well-designed genomic survey to examine the natural population structure and diversity of the European amphioxus B. lanceolatum. By applying medium-depth whole-genome sequencing of multiple individuals from two geographically separated populations (the Atlantic Ocean and the Mediterranean Sea), the authors estimated a genome-wide average heterozygosity of 2.73%, which is comparable to previous estimates for other amphioxus species and falls on the higher end of the spectrum of the known metazoan genomic heterozygosity. Little signal of population differentiation was detected between the geographically separated sampling sites, suggesting a potential model of panmixia. Based on the shorter branch length of amphioxus compared with other chordate species since their common ancestor, the authors propose that the mutation rate of amphioxus is unlikely to be high, making large effective population size a more compelling explanation for its elevated genomic diversity. Overall, I find this work interesting and well-presented. However, some of the existing analyses require more careful and rigorous execution (see below for details). In addition, it appears that the authors may have uploaded an incorrect or a very preliminary version of the supplementary tables. Please double check.

Major:

1) In this work, the authors employed bwa mem with its default setting to map the B. lanceolatum genomic reads to its reference genome. Since bwa mem relies on MEM seeding, which implicitly assumes low read-to-reference divergence. As a rule of thumb, it works really well for <2% for such read-to-reference divergence but its sensitivity quickly drops as the read-to-reference divergence level further increases (say 3%–5%). Since the average genomic heterozygosity for amphioxus also falls in 2%-5% range, the authors might want to tweak bwa mem's default parameters or used an alternative read mapper (e.g., Stampy, NextGenMap, or minimap2 with specialized settings) for more permissive mapping. Otherwise, the authors are expected to underestimate the genomic heterozygosity. This is my biggest concern for this study. In fact, this factor alone might explain the authors' finding that their estimates "are among the lowest" compared to the estimates for other amphioxus species.

We would like to thank the reviewer for this informative and relevant comment. This comment on the appropriateness of our genome mapping methods is closely related to the next comment, which concerns the appropriateness of our variant calling methods. We used a common approach to tackle both issues. This is why we have replied to this comment jointly. Please, find our response below, under the second comment.

2) Following the same logic, the default GATK-based variant calling might not be ideal, especially with BQSR. Additional adjustment such as a higher value for --max-mnp-distance. In fact, the authors might want to use more divergence-tolerant callers such as freebayes or deepvariant.

We agree that our read mapping and variant-calling pipeline was designed to prioritise specificity over sensitivity. This was an intentional methodological choice. Our aim was to minimise the risk of false-positive variant calls, even if this resulted in a more conservative estimate of genetic diversity. We believe it is better to report a lower estimate supported by high-confidence variant calls than to inflate estimates by including spurious variants.

This is particularly important in the context of species with exceptionally high levels of genetic diversity, where even a small proportion of false-positive calls could substantially bias downstream estimates. Therefore, we believe that a conservative approach provides the most reliable and scientifically rigorous basis for characterising genetic diversity. For these reasons, we remain confident that the methodological choices adopted in our original analysis are appropriate

Nevertheless, we acknowledge that different methodological approaches could affect our results. To assess the robustness of our results, we performed a methodological comparison of genome mapping and variant calling. We used an additional genome mapping tool (Minimap2) and a variant-calling tool (FreeBayes), both of which were suggested by the reviewer. The parameters used in both cases were chosen to address the reviewer's concerns and are more tolerant of divergence. In total, we compared four different method combinations: BWA MEM2 + GATK, BWA MEM2 + FreeBayes, Minimap2 + GATK, and Minimap2 + FreeBayes. To save time and computational resources, we ran this comparison on the four smallest chromosomes of the reference genome used (BraLan3). In other words, we performed a methodological comparison on chromosomes 16, 17, 18 and 19. To directly test how the different methodologies affect the main results, we reproduced Figure 1B (overall heterozygosity per sample) and Figure 2A (PCA) for each method combination, as these figures are central to our manuscript results.

The results of the methodological comparison have been incorporated into the text and are presented in Supplementary Figure 3. Overall heterozygosity appears consistent across the two variant-calling tools, though it is slightly affected by the genome mapping tool used. Interestingly, the heterozygosity resulting from calls performed after Minimap2 tends to be slightly smaller than the heterozygosity after BWA MEM2, despite the former being more flexible and allowing for more divergent mappings. We believe that our variant quality filters might have filtered out calls at sites where Minimap2 allowed for less confident mappings, but retained them in the more conservative BWA MEM2 mappings, resulting in slightly lower heterozygosity in the calls after Minimap2. Regarding the PCA, we observe the opposite pattern: the variant caller has a greater impact, while the mapper has a lower impact. In all four cases, PC1 explains a large proportion of the variance (22–15%) and is not explained by population or sex (similar to Figure 2A). PC2 in the PCAs performed after GATK are split by population (similar to Figure 2A), whereas PC2 in the PCAs performed after FreeBayes are not. Our population structure analysis suggests that population differentiation is very small and indicates a near-panmictic situation. The fact that PC2 after FreeBayes is unable to split by population is consistent with this near-panmictic scenario.

Overall, the results of the methodological comparison demonstrate the robustness of the findings in our manuscript and support the use of the methodology originally chosen. We would like to thank the reviewer for these insightful comments, which have led to a thorough analysis of our methodology and strengthened the robustness of the results in our manuscript.

3) Once the above-mentioned issues of potential mapping and variant calling biases are properly addressed, I would encourage the authors to better characterize their defined callable regions for deeper biological insights. For example, what is the chromosomal distribution of these regions? Do these regions show an enrichment for exonic regions and a depletion for repetitive sequences? Any GO term enrichment for genes captured in these regions? Do these regions also show higher levels of inter-specific conservation between different amphioxus species.

We added a new Supplementary figure and a table that characterize the callable regions (Supplementary figure 3 and Supplementary table 3). We show their chromosomal distribution, length distribution and perform an enrichment test for different genomic features (exons, introns, promoters and intergenic regions). We thank the reviewer for having pointed out that this was meaningful and missing. We believe that our manuscript has improved with these additions.

4) It would add extra value to this study if the authors could run their population differentiation and sample-based clustering analyses for nuclear and mitochondrial variants separately.

We thank the reviewer for pointing this out. In fact, there is no mitochondrial chromosome in the reference genome used, so our focus is on nuclear chromosome diversity only. The newest NCBI genome assembly (klBraLanc5.hap2) does not include a mitochondrial chromosome reference either.

In order to clarify this in the text we have added this sentence in the methods section:

> The high quality of this reference genome is essential for our analysis, but it restricts our analysis to the nuclear chromosomes since the mitochondrial chromosome is missing from the reference.

5) The current supplementary table file doesn't look right, which makes it impossible to evaluate the referenced supplementary tables in the main text. Please check carefully and make sure to upload the correct file.

We apologise for the poor formatting of the supplementary tables, which lost their labels when they were converted to PDF. This issue has now been fixed.

Minor:

1) I don't know how feasible it is and it probably goes out of the scope of genetics but it would be really cool if the authors could use ocean current data to justify their proposal that the gametes generated by the two populations can actually meet during the spawning season of B. lanceolatum.

Thanks to the reviewer's comment, we conducted further bibliographical research and added two references proving the existence of bidirectional water flow in the Strait of Gibraltar. Based on our findings, we strongly

believe that amphioxus embryos and larvae migrate through this strait at a rate that brings their population close to panmixia. We have added these two references to the discussion of our manuscript.

2) Data access. It appears to me that the provided BioProject accession id (PRJEB106885) for raw sequencing reads cannot be found in the ENA database. Please double check.
We absolutely agree with the reviewer that the raw data should be made publicly available. We are committed to making the data fully public. At the moment of the first application we were in the process of uploading the data and for this reason the ENA accession ID provided was not yet public. The raw sequencing reads from our study are now available in the ENA database under the accession number PRJEB115533. Moreover, following the suggestion of another reviewer we also made public the variant calls in our sample. The VCF file containing the calls is available in the European Variation Archive (EVA) under the project PRJEB120745 and the analysis ERZ29712263.

3) line 82: I don't think an average mapping depth of 13.26X in this study can be considered as "high-coverage whole-genome sequencing". "Medium-coverage" sounds more accurate.
Changed, thanks.

4) Figure 1D: I find this figure panel is very interesting and the underlying data should be properly presented using a supplementary table. This information is highly valuable and warrants a dedicated supplementary table.
Thank you very much for recognising the value of the data in this figure. As requested, we have added the underlying information as a supplementary table (Supplementary table 4).

5) Figure 2B: I recommend using distinct shapes to differentiate between male and female samples.
Done, thank you.

6) Figure 3: I think this figure might be too simple to justify its appearance as a single panel main figure, especially considering the fact that the shorter branch lengths of different amphioxus species have been shown in multiple published studies.
We agree with the reviewer that this figure does not provide any new information compared to those published previously. However, given the significant improvement in the quality of the amphioxus genome references, it is necessary to revalidate this figure using the new genomes. We believe that this figure could be of interest to the community, which is why we have decided to keep it in the main text rather than in the supplementary figures. We considered merging figures 2 and 3, but that does not make complete sense either. For now, we have kept the figure in the main text. However, if the reviewer and editor insist on reserving it for the supplementary data or merging figures 2 and 3, we will do so.

7) line 282: the unit of the mutation rate u needs to be provided.
Done, thank you for pointing this out.

Reviewer #3:

Overall assessment

This is a high-quality submission with rigorous data, innovative methods, and strong conclusions. It is suitable for publication in PLOS Genetics (minor revision).

The manuscript provides the first accurate quantification of genomic diversity in the European amphioxus (B. lanceolatum) using multi-individual, high-coverage whole-genome sequencing data combined with a chromosome-level reference genome (BraLan3). It reports heterozygosity of ~2.73% and π of ~2.97%, and uses population-genetic analyses plus simulations to demonstrate that this exceptionally high diversity is primarily driven by a very large effective population size (Ne ~5 million) and near-panmixia.

Highlights: the conservative "callable genome" strategy, the clear cross-species comparison figure (Fig. 1D), and complete public availability of data and code (ENA PRJEB106885 + GitHub + Zenodo).

The only significant issues are two minor inconsistencies in the data and a small amount of writing polish; everything else consists of minor technical or writing points.

Minor concerns (requiring author response/revision)

1.Sample-size inconsistency (critical): The Results section (line 90) states "38 individuals", while the Methods (page 28, line 385) and Supplementary Table 1 describe "36 adults... 9 females and 10 males... 9 females and 8 males". Please unify the number throughout the manuscript and confirm the exact count used in all analyses in Supplementary Table 1.

Thank you very much for pointing out this critical typographical error. We have corrected it, and it now states that our dataset includes 36 individuals.

2.The callable genome represents only 40.34% of the assembly (line 103). Although the conservative filtering is scientifically justified, the authors should explicitly acknowledge in the Discussion that this is a lower-bound estimate and that repetitive or paralog-rich regions (excluded by SNPable) may harbour additional diversity. Suggested addition: "we acknowledge that our estimate is a lower-bound...".

Thank you very much for this suggestion. We agree entirely with the reviewer that this should be discussed in the text. We have added the following sentences to the part of our discussion where we talk about the measured heterozygosity:

> This is a lower-bound estimate, as our analysis was limited to the callable part of the genome where heterozygosity could be accurately determined. The non-callable part of the genome contains a high proportion of repeats and duplications, and it may harbor even more heterozygosity.

3. The effective population size (Ne) estimation (Discussion, line 372) relies on the mutation rate from B.

floridae (1.41×10^{-9}). Please strengthen the caveat by explicitly describing it as a "rough approximation" and citing additional literature on mutation rates in cephalochordates.

We have modified the paragraph containing the highly approximate estimate of Ne to emphasise that it is an approximation and to explain why. We have also added another mutation rate estimate of another species of amphioxus. This part is now on Results.

4. The cross-species comparison in Fig. 1D is excellent, but the main text (line 139) should more clearly highlight the methodological distinction between "whole-genome multi-individual" versus "single-individual" estimates (the figure legend already notes this, but a reinforcing sentence in the Results or Discussion would be helpful).

We are grateful for your appreciation of the figure and agree that the text should provide further commentary on the comparison of single versus multiple individual estimates. We have added two sentences and a new reference to this effect.

Added in the Results:

> An estimate based on a single individual's whole genome is less accurate than one based on multiple individuals' whole genomes (Figure 1D).

Added in the Discussion:

> Fourth, extrapolating the genomic diversity of a single individual to the whole population depends heavily on the sampled individual's particular ancestry and can be very biased.

New reference:

> Shringarpure S, Xing EP. Effects of Sample Selection Bias on the Accuracy of Population Structure and Ancestry Inference. G3 GenesGenomesGenetics. 2014;4: 901–911. doi:10.1534/g3.113.007633

Point-by-Point comments

Title & authors (lines 1–10)

The title is clear and attractive, fully consistent with PLOS Genetics style.

The author list and corresponding-author asterisks are correct. Minor suggestion: add "(Cephalochordata)" after Branchiostoma lanceolatum for non-specialist readers.

Thank you for the suggestion. We have added "(Cephalochordata)" to the abstract to provide context to the non-specialists while keeping the title short and clear.

Abstract (lines 12–24)

Excellent: logical flow, prominent data (2.73% heterozygosity), and strong conclusions.

Line 18: "average heterozygosity of 2.73%" – this differs slightly from the main text (lines 108–109: 2.74% Atlantic / 2.72% Mediterranean). Please harmonise to "~2.73%".

Thank you; we have now amended the Results section.

Line 22: "near-panmictic" – perfect.
Author summary (lines 26–37)
Clear, accessible, and well suited to PLOS Genetics.
Line 30: "some of the highest levels" → change to "among the highest reported in animals" for greater precision.

Done, thank you.

Introduction (lines 42–86)
Lines 44–45: citation [1] is well chosen.
Lines 60–62: historical literature review is accurate.
Lines 74–78: the $\theta = 4Ne\mu$ explanation is correct.
Lines 84–86: the concluding sentence is powerful.
Minor (optional):
Line 53 "sister-group to vertebrates and tunicates" → consider updating to "sister group to Olfactores (vertebrates + tunicates)" to reflect current phylogenomic consensus.

Done, thank you.

Results – Genomic diversity (lines 88–125)
Line 90: sample number (38 vs 36) – must be corrected.

Done, thank you.

Lines 102–104: the callable-genome definition (SNPable + depth ≥5× + pruning) is exceptionally rigorous and well explained – excellent.
Lines 108–110: heterozygosity values and the non-significant t-test result are clearly presented.
Lines 114–119: the Fisher exact test results for exons/introns/promoters versus intergenic regions are convincing and nicely illustrate purifying selection.
Lines 121–124: the high proportion of multi-allelic variants (11.99% SNPs, 58% INDELs) is a key observation and appropriately highlights departure from the infinite-sites model.
Fig. 1 legend (lines 126–135): complete and clear. Fig. 1D is one of the strongest visual elements of the paper.
Results – Population differentiation (lines 150–180+, including Fig. 2)
Line 154: FST = 0.00429 is correctly interpreted as extremely low.
Lines 159–169: the PCA (with multiple subsets and allele-frequency stratification) is comprehensive and strongly supports gene flow.
Lines 171–177: the distance tree is concise and informative.
Lines 179–187 (page 16): the shared/private variants permutation test is elegantly designed.
Fig. 2 legend: clear.
Later results (phylogeny + simulations, ~lines 245–290, Figs 3–4)
Fig. 3 tree construction (one-to-one orthologs + IQ-TREE) is rigorous.
Fig. 4 simulation framework (high Ne vs high μ, with PCA on the site-frequency spectrum) is clever and provides critical evidence.
Minor: At the Ne estimate (line 372), add "assuming μ similar to B. floridae" for clarity.

Done, thank you.

Discussion (lines 293–382)

Lines 293–305: framing amphioxus as an evo-devo model and contrasting "phenotypic simplicity vs genomic complexity" is excellent.

Lines 306–332: the discussion of methodological biases in diversity estimates (few loci vs WGS, single vs multi-individual) is one of the most insightful in the literature.

Lines 346–351: the brief mention of meiotic consequences is appropriately cautious.

Lines 354–363: the contrast between multi-allelic sites and panmixia is brilliant.

Lines 364–374: the Ne estimation using B. floridae μ plus explicit caveats is honest and transparent.

Closing paragraph (lines 379–382): the link from larval dispersal to panmixia is logically tight.

Minor: Line 373 Ne value "5,267,730" → change to "approximately 5.3 million" for readability.

Done, thank you.

Methods (lines 384–487+)

Sampling & sequencing: detailed and reproducible.

Variant calling: GATK best practices + two rounds of BQSR + three-layer hard filtering + SNPable c=3 are extremely rigorous (Supplementary Fig. 1 workflow is perfect).

Additional filters (lines 425–444): the depth 5–20× + 20% window pruning approach is ingenious for excluding CNVs and isolated variants.

All analysis scripts (Snakemake) + Zenodo frozen version → full reproducibility score.

Minor: Line 385 "36" conflicts with Results → please unify.

Done, thank you.

Data availability & Other sections

Perfect: ENA accession + GitHub + Zenodo.

Competing Interests / Funding / Government Employee statements fully comply with journal requirements.

Supplementary materials

Supplementary Figs 1–4 and Tables 1–5 are clear and supportive.

Suggested revision priorities

Immediately correct the sample-size inconsistency (38 vs 36).

Harmonise the heterozygosity value (~2.73%).

Add one sentence in the Discussion acknowledging the lower-bound nature of the callable-genome estimate.

Minor English polishing of a few long sentences (e.g., lines 321–332).

I recommend acceptance after minor revision. This manuscript will be an outstanding contribution to our understanding of extreme genomic diversity and larval-mediated panmixia in marine invertebrates.

We addressed all of the suggested revision priorities and smaller comments. Thank you for appreciating our work.

Reviewer #4:

This study provides the first high-quality assessment of genomic diversity in the European amphioxus (Branchiostoma lanceolatum), revealing exceptionally high levels of genetic variation, with an average heterozygosity of approximately 3%. Despite sampling individuals from geographically distant Atlantic and Mediterranean locations, the research found minimal population differentiation, indicating extensive gene flow across a broad range. Phylogenetic and population genetic simulations demonstrate that this elevated genomic diversity is primarily driven by a very large effective population size. The authors conclude that long-distance larval dispersal via ocean currents sustains a near-panmictic population structure in this species. While the results are interesting, several aspects could be improved before publication.

Major Concerns:

1. Supporting the "near-panmictic" claim: To substantiate the claim of a "near-panmictic population structure," it would be informative to conduct standard population structure analyses using software like ADMIXTURE or STRUCTURE. Furthermore, if feasible, estimating migration rates (m) between the two populations would be highly relevant. A migration rate (m) close to 0.5 would strongly support a panmictic scenario.

We are very grateful to the reviewer for this comment. It has led us to add two very valuable results to our manuscript that make the results more robust.

We ran ADMIXTURE analysis on our dataset. Its results show that the optimal K for our samples is K=1 supporting the near-panmictic context shown in other population structure analysis.

In addition, following the reviewer's recommendation, we applied an island model to our two sampling sites in order to calculate the theoretical migration rate and the number of migrants needed per generation to maintain the near-panmictic situation observed in our data. When calculating the migration rate, a series of assumptions are made and a mutation rate measured in a sister species is used, which makes the resulting migration rate estimate very rough and meant to provide a general idea rather than an exact estimation. The calculated migration rate is $4.6 \cdot 10^{-5}$, resulting in an estimated number of migrants between populations per generation required to maintain the observed near-panmictic situation of 58. Although these results are not exact, they suggest that, given the enormous population size of *B. lanceolatum* and its consequent high genomic diversity, little migration is necessary between populations every generation to maintain the near-panmictic situation.

The addition of these analysis to our manuscript makes our near-panmixia results more robust.

2. Validating the diversity estimate: The claim of providing an "unprecedentedly accurate estimate" (Line 305) is not fully substantiated. The high heterozygosity and large divergence between the reference genome and sequenced individuals can introduce false positives or false negatives in variant calling, an issue not systematically evaluated. As highlighted in the mutation rate study by Xue et al. (2026), significant sequence divergence can

lead to a high rate of misaligned reads. The authors themselves note their estimates are a "lower-bound" of true diversity. To address this, analyses (e.g., simulations) should be performed to evaluate the accuracy of the pipeline, or the claim should be toned down throughout the manuscript.

The sample used to construct the BraLan3 reference genome, which was used in this study, originated from Banyuls. Our population structure analyses revealed minimal divergence between the Mediterranean and Atlantic populations. Consequently, we expect the genetic divergence between our samples and the reference genome to be low, so we do not consider reference bias to be a major concern for genome mapping. However, we acknowledge that the exceptionally high genetic diversity of *B. lanceolatum* results in significant sequence variation among individuals, even within the same population. This variation makes read mapping more challenging and increases the potential for erroneous alignments.

We designed our read mapping and variant-calling pipeline to prioritise specificity over sensitivity in view of these mapping challenges. Our aim was to minimise the risk of false-positive variant calls, even if this resulted in a more conservative estimate of genetic diversity. We believe that reporting a lower estimate supported by high-confidence variant calls is preferable to inflating diversity estimates by including spurious variants. For these reasons, we remain confident that the methodological choices adopted in our original analysis are appropriate. However, we acknowledge that the mapping challenges associated with such high levels of genetic diversity may have influenced some of our results. Therefore, we have moderated our claims regarding the accuracy of our estimates throughout the manuscript.

Following a suggestion by reviewer #2 and in order to evaluate whether these mapping challenges could have affected our conclusions as raised by this reviewer, we performed additional analyses. Specifically, we compared our original, more conservative genome mapping and variant-calling pipeline with alternative, more divergence-tolerant methods. We evaluated an additional genome mapping tool (Minimap2) and an alternative variant caller (FreeBayes), resulting in four methodological combinations: BWA-MEM2 + GATK, BWA-MEM2 + FreeBayes, Minimap2 + GATK and Minimap2 + FreeBayes. To reduce computational time and resource requirements, this comparison was performed on chromosomes 16, 17 ,18 and 19 of the BraLan3 reference genome. To assess the impact of different methodological choices on our study's main findings, we reproduced Figures 1B (overall heterozygosity per sample) and 2A (principal component analysis) for each combination of methods.

The results of the methodological comparison have been incorporated into the text and are presented in Supplementary Figure 3. Overall heterozygosity appears consistent across the two variant-calling tools, though it is slightly affected by the genome mapping tool used. Interestingly, the heterozygosity resulting from calls performed after Minimap2 tends to be slightly smaller than the heterozygosity after BWA MEM2, despite the former being more flexible and allowing for more divergent mappings. We believe that our variant quality filters might have filtered out calls at sites where Minimap2 allowed for less confident mappings, but retained them in the more conservative BWA MEM2 mappings, resulting in slightly lower heterozygosity in the calls after Minimap2. Regarding the PCA, we observe the opposite pattern: the variant caller has a greater impact, while the mapper has a lower impact. In all four cases, PC1 explains a large proportion of the variance (22–15%) and is

not explained by population or sex (similar to Figure 2A). PC2 in the PCAs performed after GATK split by population (similar to Figure 2A), whereas PC2 in the PCAs performed after FreeBayes do not. Our population structure analysis suggests that population differentiation is very small and indicates a near-panmictic situation. The fact that PC2 after FreeBayes is unable to split by population is consistent with this near-panmictic scenario.

Overall, the methodological comparison shows that the results of our study are robust to the use of more flexible and divergence-tolerant genome mapping and variant-calling approaches. We thank the reviewer for this valuable suggestion, which led us to conduct a comprehensive review of our methodology, thereby strengthening the reliability of the results presented in this study.

3. Supplementary materials: The supplementary tables lack numbering, which appears to be an oversight and should be corrected.

We apologise for the poor formatting of the supplementary tables, which lost their labels when they were converted to PDF. This issue has now been fixed.

Minor Concerns:

1. Figure 2A: The labels within the PCA plot dots are too small and unnecessary. They should be removed for clarity.

Done, thank you.

2. Lines 216-218: The assertion that external fertilization results in "pairings that are close to random" compared to internal fertilization is not self-evident. A more detailed explanation is warranted.

Thank you for raising this issue. We have extended the corresponding explanation and added a new reference to support our claims.

> In external fertilization, natural selection acts only after gamete release, through sperm competition and gamete-level mate choice. In contrast, internal fertilization involves stronger pre-mating sexual selection, resulting in greater deviation from random mating.

Added reference:

> Evans JP, Lymbery RA. Sexual selection after gamete release in broadcast spawning invertebrates. Philos Trans R Soc B Biol Sci. 2020;375: 20200069. doi:10.1098/rstb.2020.0069

3. Lines 323-325: The statement that increasing sequencing depth can overcome challenges related to incorrectly mapped reads is misleading. Higher depth does not inherently resolve mapping errors. This point should be revised or supported with simulation data.

We appreciate the reviewer highlighting the ambiguity in our explanation of this matter. We have rephrased the paragraph and added a new reference to make it clearer and more precise.

The presence of high genomic diversity makes estimation more challenging. In these cases, the sequencing reads differ more from the genome reference, making them more difficult to map accurately. This challenge can be partially overcome by increasing sequencing depth and restricting analysis to more easily mappable parts of the genome. These precautions allow for higher-quality heterozygote and homozygote calls, compensate for random sequencing errors, and reduce incorrectly mapped reads.

Added reference:

Sims D, Sudbery I, Ilott NE, Heger A, Ponting CP. Sequencing depth and coverage: key considerations in genomic analyses. Nat Rev Genet. 2014;15: 121–132. doi:10.1038/nrg3642

4. Line 367: The cited mutation rate (μ) value appears incorrect. According to the referenced paper, it should be 5.89×10^{-9}, and the subsequent effective population size (Ne) estimate for B. lanceolatum should be recalculated.

We are very thankful to the reviewer for pointing this out. Thanks to their comment, we realized that the mutation rate in the reference changed between the preprint (which we had based ourselves on originally) and the final publication. We have updated the mutation rate in our calculations.

5. Line 446 ("Genomic diversity analysis"): The methods description in this section is too brief. More detailed steps should be provided.

We agree that this section of the methods needed more detail. Thank you for pointing this out. We have now expanded it to be clearer and more precise.

We used VariantsToTable to extract genotypes from the VCF file. Then we used in-house scripts (see Data and code availability) to calculate heterozygosity per sample, nucleotide diversity (π), and the number of alleles per variant. We calculated heterozygosity per sample by counting the number of heterozygous sites in a given region of the genome and dividing it by the length of that region. Total heterozygosity for a given sample, for example, is equal to the total number of heterozygous sites of that sample in the callable genome divided by the length of the callable genome. Nucleotide diversity, or the average pairwise differences, was calculated for a group of samples (either all Atlantic, all Mediterranean, or all samples) and normalized by sequence length. We used the following formula:

$$\pi = \frac{\frac{\sum_{i<j} d_{ij}}{p}}{L}$$

L corresponds to the length of the region in question, p corresponds to the number of pairwise comparisons and dij corresponds to the number of nucleotide differences between sequences i and j in the region in question. Using the gene annotation from BraLan3, we divided the genome into exons, introns, promoters (1 kbp upstream of all genes), and intergenic regions. We then evaluated heterozygosity and nucleotide diversity in the total length of the callable genome, as well as in each of these groups of regions.
