# Supplementary material for: The highly heterozygous European amphioxus (Branchiostoma lanceolatum) at the edge of panmixia
Source: PLoS Genet. 2026 Sep 8;22(9):e1012293. doi: 10.1371/journal.pgen.1012293 (PMC13577491; doi:10.1371/journal.pgen.1012293)
Supplement: Supplementary file 2 [file pgen.1012293.s017.pdf]

Response to Reviewers

Reviewer #2:

The authors have thoroughly addressed my previous concerns and comments. I think these additional analyses further enhanced the depth of this manuscript.

However, there is a significant typesetting error that must be corrected prior to publication:
Many of the cited references are mismatched between the main text and the reference section. For instance, references 61–67 are cited regarding amphioxus mutation rates, but entries 61–67 in the reference list are completely unrelated. Please perform a comprehensive check of all references to ensure the in-text citations match the reference list correctly.

We apologize for these typesetting errors and thank the reviewer for pointing them out. The reference list has been updated and revised thoroughly.

Reviewer #4:

The authors have addressed all my comments and I have no further comment.
