# Supplementary material for: The highly heterozygous European amphioxus (Branchiostoma lanceolatum) at the edge of panmixia
Source: PLoS Genet. 2026 Sep 8;22(9):e1012293. doi: 10.1371/journal.pgen.1012293 (PMC13577491; doi:10.1371/journal.pgen.1012293)
Supplement: Peer review history

# Peer review: Decision Letter 0

**DOI:** 10.1371/journal.pgen.1012293.r001

**Authors:** Justin Fay, Jae Young Choi

26 Mar 2026

PGENETICS-D-26-00218

The highly heterozygous European amphioxus (*Branchiostoma lanceolatum* ) at the edge of panmixia

PLOS Genetics

Dear Dr. Brasó-Vives,

Thank you for submitting your manuscript to PLOS Genetics. After careful consideration, we feel that it has merit but does not fully meet PLOS Genetics's publication criteria as it currently stands. Therefore, we invite you to submit a revised version of the manuscript that addresses the points raised during the review process.

Please submit your revised manuscript by May 25 2026 11:59PM. If you will need more time than this to complete your revisions, please reply to this message or contact the journal office at plosgenetics@plos.org. When you're ready to submit your revision, log on to https://www.editorialmanager.com/pgenetics/ and select the 'Submissions Needing Revision' folder to locate your manuscript file.

Please include the following items when submitting your revised manuscript:

* A letter that responds to each point raised by the editor and reviewer(s). You should upload this letter as a separate file labeled 'Response to Reviewers'. This file does not need to include responses to any formatting updates and technical items listed in the 'Journal Requirements' section below.

* A marked-up copy of your manuscript that highlights changes made to the original version. You should upload this as a separate file labeled 'Revised Manuscript with Track Changes'.

* An unmarked version of your revised paper without tracked changes. You should upload this as a separate file labeled 'Manuscript'.

If you would like to make changes to your financial disclosure, competing interests statement, or data availability statement, please make these updates within the submission form at the time of resubmission. Guidelines for resubmitting your figure files are available below the reviewer comments at the end of this letter.

We look forward to receiving your revised manuscript.

Kind regards,

Jae Young Choi

Academic Editor

PLOS Genetics

Justin Fay

Section Editor

PLOS Genetics

Aimée Dudley

Editor-in-Chief

PLOS Genetics

Anne Goriely

Editor-in-Chief

PLOS Genetics

**Additional Editor Comments:**

The manuscript has been reviewed by four reviewers. The reviewers have found the topic of the study and results interesting, however they also had some concerns about the current manuscript. Primary concerns were relating to technical analysis of the genomic data (reviewers 2 and 3) and the evolutionary analysis (reviewer 2 and 4). There were also several comments where clarity could be greatly improved and issues with the labeling of the supplemental materials. In addition multiple reviewers have commented on the availability of the underlying dataset for this study. In the end we think addressing these issues warrants a major revision. Based on the reviews, we will not be able to accept this version of the manuscript, but we would be willing to review a much-revised version that fully addresses each of the points raised by the reviewers.

**Journal Requirements:**

If the reviewer comments include a recommendation to cite specific previously published works, please review and evaluate these publications to determine whether they are relevant and should be cited. There is no requirement to cite these works unless the editor has indicated otherwise.

1) We ask that a manuscript source file is provided at Revision. Please upload your manuscript file as a .doc, .docx, .rtf or .tex. If you are providing a .tex file, please upload it under the item type u2018LaTeX Source Fileu2019 and leave your .pdf version as the item type u2018Manuscriptu2019.

2) Please upload all main figures as separate Figure files in .tif or .eps format. For more information about how to convert and format your figure files please see our guidelines:

https://journals.plos.org/plosgenetics/s/figures

3) We have noticed that you have a list of Supporting Information legends in your manuscript. However, there are no corresponding files uploaded to the submission. Please upload them as separate files with the item type 'Supporting Information'.

4) We notice that your supplementary Figures are included in the manuscript file. Please remove them and upload them with the file type 'Supporting Information'. Please ensure that each Supporting Information file has a legend listed in the manuscript after the references list.

5) In the online submission form, you indicated that your data will be submitted to a repository upon acceptance. We strongly recommend all authors deposit their data before acceptance, as the process can be lengthy and hold up publication timelines. Please note that, though access restrictions are acceptable now, your entire minimal dataset will need to be made freely accessible if your manuscript is accepted for publication. This policy applies to all data except where public deposition would breach compliance with the protocol approved by your research ethics board. If you are unable to adhere to our open data policy, please kindly revise your statement to explain your reasoning and we will seek the editor's input on an exemption.

6) Please amend your detailed Financial Disclosure statement. This is published with the article. It must therefore be completed in full sentences and contain the exact wording you wish to be published.

1) State what role the funders took in the study. If the funders had no role in your study, please state: "The funders had no role in study design, data collection and analysis, decision to publish, or preparation of the manuscript."

2) If any authors received a salary from any of your funders, please state which authors and which funders..

If you did not receive any funding for this study, please simply state: u201cThe authors received no specific funding for this work.u201d

**Reviewers' comments:**

Reviewer's Responses to Questions

**Comments to the Authors:**

**Please note here if the review is uploaded as an attachment.**

Reviewer #1: The authors provide an analysis of the genetic diversity of European amphioxus across two populations in the waters of France. They confirm the striking levels of heterozygosity that have previously been reported for various species of amphioxus and go on to provide some careful analyses that demonstrate this animal appears to exhibit panmixis, even across these distant populations. The manuscript is very clearly written and the importance of the work clearly justified. This establishes amphioxus as an extremely widespread marine species that serves as an ideal study system for understanding the evolutionary dynamics and mechanisms underpinning genetic diversity. The approach described by the authors in this study can be taken as the ‘gold standard’ for how genetic diversity should be measured in a species, with implications well beyond amphioxus research.

I find myself in the pleasurable (and highly unusual) position of not having any substantive questions or recommendations for revisions, since this is such a well-written manuscript and the analyses are thorough and clearly explained.

My only comment is whether each of the individual genome assemblies will be available in the data release? These will be extremely useful to the community, particularly given the high genetic diversity in this species, as the assemblies can be used to help locate functional and constrained genomic features. However, my reading of the data availability statement makes me think that the authors plan to only release the raw sequence reads. This would be a missed opportunity, in my view.

There are also a very small number of typos, as follows.

The authors say they have analysed Ciona intestinalis, but if they have used the KH assembly then I think that they mean that they have used Ciona robusta (even though the database itself still refers to the old naming of this species as intestinalis).

Line 411: “This is we split” does not make sense and needs amending.

Line 430: change “Shortly” to “Briefly”.

Reviewer #2: In the manuscript entitled "The highly heterozygous European amphioxus (Branchiostoma lanceolatum) at the edge of panmixia", the authors present a well-designed genomic survey to examine the natural population structure and diversity of the European amphioxus B. lanceolatum. By applying medium-depth whole-genome sequencing of multiple individuals from two geographically separated populations (the Atlantic Ocean and the Mediterranean Sea), the authors estimated a genome-wide average heterozygosity of 2.73%, which is comparable to previous estimates for other amphioxus species and falls on the higher end of the spectrum of the known metazoan genomic heterozygosity. Little signal of population differentiation was detected between the geographically separated sampling sites, suggesting a potential model of panmixia. Based on the shorter branch length of amphioxus compared with other chordate species since their common ancestor, the authors propose that the mutation rate of amphioxus is unlikely to be high, making large effective population size a more compelling explanation for its elevated genomic diversity. Overall, I find this work interesting and well-presented. However, some of the existing analyses require more careful and rigorous execution (see below for details). In addition, it appears that the authors may have uploaded an incorrect or a very preliminary version of the supplementary tables. Please double check.

Major:

1) In this work, the authors employed bwa mem with its default setting to map the B. lanceolatum genomic reads to its reference genome. Since bwa mem relies on MEM seeding, which implicitly assumes low read-to-reference divergence. As a rule of thumb, it works really well for <2% for such read-to-reference divergence but its sensitivity quickly drops as the read-to-reference divergence level further increases (say 3%–5%). Since the average genomic heterozygosity for amphioxus also falls in 2%-5% range, the authors might want to tweak bwa mem's default parameters or used an alternative read mapper (e.g., Stampy, NextGenMap, or minimap2 with specialized settings) for more permissive mapping. Otherwise, the authors are expected to underestimate the genomic heterozygosity. This is my biggest concern for this study. In fact, this factor alone might explain the authors' finding that their estimates "are among the lowest" compared to the estimates for other amphioxus species.

2) Following the same logic, the default GATK-based variant calling might not be ideal, especially with BQSR. Additional adjustment such as a higher value for --max-mnp-distance. In fact, the authors might want to use more divergence-tolerant callers such as freebayes or deepvariant.

3) Once the above-mentioned issues of potential mapping and variant calling biases are properly addressed, I would encourage the authors to better characterize their defined callable regions for deeper biological insights. For example, what is the chromosomal distribution of these regions? Do these regions show an enrichment for exonic regions and an depletion for repetitive sequences? Any GO term enrichment for genes captured in these regions? Do these regions also show higher level of inter-specific conservation between different amphioxus species.

4) It would add extra value to this study if the authors could run their population differentiation and sample-based clustering analyses for nuclear and mitochondrial variants separately.

5) The current supplementary table file doesn't look right, which makes it impossible to evaluate the referenced supplementary tables in the main text. Please check carefully and make sure to upload the correct file.

Minor:

1) I don't know how feasible it is and it probably goes out of the scope of genetics but it would be really cool if the authors could use ocean current data to justify their proposal that the gametes generated by the two populations can actually meet during the spawning season of B. lanceolatum.

2) Data access. It appears to me that the provided BioProject accession id (PRJEB106885) for raw sequencing reads cannot be found in the ENA database. Please double check.

3) line 82: I don't think an average mapping depth of 13.26X in this study can be considered as "high-coverage whole-genome sequencing". "Medium-coverage" sounds more accurate.

4) Figure 1D: I find this figure panel is very interesting and the underlying data should be properly presented using a supplementary table. This information is highly valuable and warrants a dedicated supplementary table.

5) Figure 2B: I recommend using distinct shapes to differentiate between male and female samples.

6) Figure 3: I think this figure might be too simple to justify its appearance as a single panel main figure, especially considering the fact that the shorter branch lengths of different amphioxus species have been shown in multiple published studies.

7) line 282: the unit of the mutation rate u needs to be provided.

Reviewer #3: Overall assessment

This is a high-quality submission with rigorous data, innovative methods, and strong conclusions. It is suitable for publication in PLOS Genetics (minor revision).

The manuscript provides the first accurate quantification of genomic diversity in the European amphioxus (B. lanceolatum) using multi-individual, high-coverage whole-genome sequencing data combined with a chromosome-level reference genome (BraLan3). It reports heterozygosity of ~2.73% and π of ~2.97%, and uses population-genetic analyses plus simulations to demonstrate that this exceptionally high diversity is primarily driven by a very large effective population size (Ne ~5 million) and near-panmixia.

Highlights: the conservative “callable genome” strategy, the clear cross-species comparison figure (Fig. 1D), and complete public availability of data and code (ENA PRJEB106885 + GitHub + Zenodo).

The only significant issues are two minor inconsistencies in the data and a small amount of writing polish; everything else consists of minor technical or writing points.

Minor concerns (requiring author response/revision)

1.Sample-size inconsistency (critical): The Results section (line 90) states “38 individuals”, while the Methods (page 28, line 385) and Supplementary Table 1 describe “36 adults… 9 females and 10 males… 9 females and 8 males”. Please unify the number throughout the manuscript and confirm the exact count used in all analyses in Supplementary Table 1.

2.The callable genome represents only 40.34% of the assembly (line 103). Although the conservative filtering is scientifically justified, the authors should explicitly acknowledge in the Discussion that this is a lower-bound estimate and that repetitive or paralog-rich regions (excluded by SNPable) may harbour additional diversity. Suggested addition: “we acknowledge that our estimate is a lower-bound…”.

3.The effective population size (Ne) estimation (Discussion, line 372) relies on the mutation rate from B. floridae (1.41 × 10⁻⁹). Please strengthen the caveat by explicitly describing it as a “rough approximation” and citing additional literature on mutation rates in cephalochordates.

4. The cross-species comparison in Fig. 1D is excellent, but the main text (line 139) should more clearly highlight the methodological distinction between “whole-genome multi-individual” versus “single-individual” estimates (the figure legend already notes this, but a reinforcing sentence in the Results or Discussion would be helpful).

Point-by-Point comments

Title & authors (lines 1–10)

The title is clear and attractive, fully consistent with PLOS Genetics style.

The author list and corresponding-author asterisks are correct. Minor suggestion: add “(Cephalochordata)” after Branchiostoma lanceolatum for non-specialist readers.

Abstract (lines 12–24)

Excellent: logical flow, prominent data (2.73% heterozygosity), and strong conclusions.

Line 18: “average heterozygosity of 2.73%” – this differs slightly from the main text (lines 108–109: 2.74% Atlantic / 2.72% Mediterranean). Please harmonise to “~2.73%”.

Line 22: “near-panmictic” – perfect.

Author summary (lines 26–37)

Clear, accessible, and well suited to PLOS Genetics.

Line 30: “some of the highest levels” → change to “among the highest reported in animals” for greater precision.

Introduction (lines 42–86)

Lines 44–45: citation [1] is well chosen.

Lines 60–62: historical literature review is accurate.

Lines 74–78: the θ = 4Neμ explanation is correct.

Lines 84–86: the concluding sentence is powerful.

Minor (optional):

Line 53 “sister-group to vertebrates and tunicates” → consider updating to “sister group to Olfactores (vertebrates + tunicates)” to reflect current phylogenomic consensus.

Results – Genomic diversity (lines 88–125)

Line 90: sample number (38 vs 36) – must be corrected.

Lines 102–104: the callable-genome definition (SNPable + depth ≥5× + pruning) is exceptionally rigorous and well explained – excellent.

Lines 108–110: heterozygosity values and the non-significant t-test result are clearly presented.

Lines 114–119: the Fisher exact test results for exons/introns/promoters versus intergenic regions are convincing and nicely illustrate purifying selection.

Lines 121–124: the high proportion of multi-allelic variants (11.99% SNPs, 58% INDELs) is a key observation and appropriately highlights departure from the infinite-sites model.

Fig. 1 legend (lines 126–135): complete and clear. Fig. 1D is one of the strongest visual elements of the paper.

Results – Population differentiation (lines 150–180+, including Fig. 2)

Line 154: FST = 0.00429 is correctly interpreted as extremely low.

Lines 159–169: the PCA (with multiple subsets and allele-frequency stratification) is comprehensive and strongly supports gene flow.

Lines 171–177: the distance tree is concise and informative.

Lines 179–187 (page 16): the shared/private variants permutation test is elegantly designed.

Fig. 2 legend: clear.

Later results (phylogeny + simulations, ~lines 245–290, Figs 3–4)

Fig. 3 tree construction (one-to-one orthologs + IQ-TREE) is rigorous.

Fig. 4 simulation framework (high Ne vs high μ, with PCA on the site-frequency spectrum) is clever and provides critical evidence.

Minor: At the Ne estimate (line 372), add “assuming μ similar to B. floridae” for clarity.

Discussion (lines 293–382)

Lines 293–305: framing amphioxus as an evo-devo model and contrasting “phenotypic simplicity vs genomic complexity” is excellent.

Lines 306–332: the discussion of methodological biases in diversity estimates (few loci vs WGS, single vs multi-individual) is one of the most insightful in the literature.

Lines 346–351: the brief mention of meiotic consequences is appropriately cautious.

Lines 354–363: the contrast between multi-allelic sites and panmixia is brilliant.

Lines 364–374: the Ne estimation using B. floridae μ plus explicit caveats is honest and transparent.

Closing paragraph (lines 379–382): the link from larval dispersal to panmixia is logically tight.

Minor: Line 373 Ne value “5,267,730” → change to “approximately 5.3 million” for readability.

Methods (lines 384–487+)

Sampling & sequencing: detailed and reproducible.

Variant calling: GATK best practices + two rounds of BQSR + three-layer hard filtering + SNPable c=3 are extremely rigorous (Supplementary Fig. 1 workflow is perfect).

Additional filters (lines 425–444): the depth 5–20× + 20% window pruning approach is ingenious for excluding CNVs and isolated variants.

All analysis scripts (Snakemake) + Zenodo frozen version → full reproducibility score.

Minor: Line 385 “36” conflicts with Results → please unify.

Data availability & Other sections

Perfect: ENA accession + GitHub + Zenodo.

Competing Interests / Funding / Government Employee statements fully comply with journal requirements.

Supplementary materials

Supplementary Figs 1–4 and Tables 1–5 are clear and supportive.

Suggested revision priorities

Immediately correct the sample-size inconsistency (38 vs 36).

Harmonise the heterozygosity value (~2.73%).

Add one sentence in the Discussion acknowledging the lower-bound nature of the callable-genome estimate.

Minor English polishing of a few long sentences (e.g., lines 321–332).

I recommend acceptance after minor revision. This manuscript will be an outstanding contribution to our understanding of extreme genomic diversity and larval-mediated panmixia in marine invertebrates.

Reviewer #4: This study provides the first high-quality assessment of genomic diversity in the European amphioxus (Branchiostoma lanceolatum), revealing exceptionally high levels of genetic variation, with an average heterozygosity of approximately 3%. Despite sampling individuals from geographically distant Atlantic and Mediterranean locations, the research found minimal population differentiation, indicating extensive gene flow across a broad range. Phylogenetic and population genetic simulations demonstrate that this elevated genomic diversity is primarily driven by a very large effective population size. The authors conclude that long-distance larval dispersal via ocean currents sustains a near-panmictic population structure in this species. While the results are interesting, several aspects could be improved before publication.

Major Concerns:

1. Supporting the "near-panmictic" claim: To substantiate the claim of a "near-panmictic population structure," it would be informative to conduct standard population structure analyses using software like ADMIXTURE or STRUCTURE. Furthermore, if feasible, estimating migration rates (m) between the two populations would be highly relevant. A migration rate (m) close to 0.5 would strongly support a panmictic scenario.

2. Validating the diversity estimate: The claim of providing an "unprecedentedly accurate estimate" (Line 305) is not fully substantiated. The high heterozygosity and large divergence between the reference genome and sequenced individuals can introduce false positives or false negatives in variant calling, an issue not systematically evaluated. As highlighted in the mutation rate study by Xue et al. (2026), significant sequence divergence can lead to a high rate of misaligned reads. The authors themselves note their estimates are a "lower-bound" of true diversity. To address this, analyses (e.g., simulations) should be performed to evaluate the accuracy of the pipeline, or the claim should be toned down throughout the manuscript.

3. Supplementary materials: The supplementary tables lack numbering, which appears to be an oversight and should be corrected.

Minor Concerns:

1. Figure 2A: The labels within the PCA plot dots are too small and unnecessary. They should be removed for clarity.

2. Lines 216-218: The assertion that external fertilization results in "pairings that are close to random" compared to internal fertilization is not self-evident. A more detailed explanation is warranted.

3. Lines 323-325: The statement that increasing sequencing depth can overcome challenges related to incorrectly mapped reads is misleading. Higher depth does not inherently resolve mapping errors. This point should be revised or supported with simulation data.

4. Line 367: The cited mutation rate (μ) value appears incorrect. According to the referenced paper, it should be 5.89 × 10⁻⁹, and the subsequent effective population size (Ne) estimate for B. lanceolatumshould be recalculated.

5. Line 446 ("Genomic diversity analysis"): The methods description in this section is too brief. More detailed steps should be provided.

**********

**Have all data underlying the figures and results presented in the manuscript been provided?**

Large-scale datasets should be made available via a public repository as described in the *PLOS Genetics*  data availability policy, and numerical data that underlies graphs or summary statistics should be provided in spreadsheet form as supporting information.

Reviewer #1: Yes

Reviewer #2: **No:** See my comments for the current supplementary table file.

Reviewer #3: Yes

Reviewer #4: Yes

**********

PLOS authors have the option to publish the peer review history of their article (what does this mean?). If published, this will include your full peer review and any attached files.

If you choose “no”, your identity will remain anonymous but your review may still be made public.

**Do you want your identity to be public for this peer review?**  For information about this choice, including consent withdrawal, please see our Privacy Policy.

Reviewer #1: No

Reviewer #2: No

Reviewer #3: No

Reviewer #4: No

**Figure resubmission:**

While revising your submission, we strongly recommend that you use PLOS’s NAAS tool (https://ngplosjournals.pagemajik.ai/artanalysis) to test your figure files. NAAS can convert your figure files to the TIFF file type and meet basic requirements (such as print size, resolution), or provide you with a report on issues that do not meet our requirements and that NAAS cannot fix.

After uploading your figures to PLOS’s NAAS tool - https://ngplosjournals.pagemajik.ai/artanalysis, NAAS will process the files provided and display the results in the "Uploaded Files" section of the page as the processing is complete. If the uploaded figures meet our requirements (or NAAS is able to fix the files to meet our requirements), the figure will be marked as "fixed" above. If NAAS is unable to fix the files, a red "failed" label will appear above. When NAAS has confirmed that the figure files meet our requirements, please download the file via the download option, and include these NAAS processed figure files when submitting your revised manuscript.

**Reproducibility:**

To enhance the reproducibility of your results, we recommend that authors of applicable studies deposit laboratory protocols in protocols.io, where a protocol can be assigned its own identifier (DOI) such that it can be cited independently in the future. Additionally, PLOS ONE offers an option to publish peer-reviewed clinical study protocols. Read more information on sharing protocols at https://plos.org/protocols?utm_medium=editorial-email&utm_source=authorletters&utm_campaign=protocols

# Author comment: Author response to Decision Letter 1

**DOI:** 10.1371/journal.pgen.1012293.r002

31 Jul 2026

Attachment Submitted filename: MBrasoVives_etal_Response_to_Reviewers.pdf

# Peer review: Decision Letter 1

**DOI:** 10.1371/journal.pgen.1012293.r003

**Authors:** Justin Fay, Jae Young Choi

10 Aug 2026

PGENETICS-D-26-00218R1

The highly heterozygous European amphioxus (*Branchiostoma lanceolatum* ) at the edge of panmixia

PLOS Genetics

Dear Dr. Brasó-Vives,

Thank you for submitting your manuscript to PLOS Genetics. After careful consideration, we feel that it has merit but does not fully meet PLOS Genetics's publication criteria as it currently stands. Therefore, we invite you to submit a revised version of the manuscript that addresses the points raised during the review process.

Please submit your revised manuscript within by Sep 09 2026 11:59PM. If you will need more time than this to complete your revisions, please reply to this message or contact the journal office at plosgenetics@plos.org. When you're ready to submit your revision, log on to https://www.editorialmanager.com/pgenetics/ and select the 'Submissions Needing Revision' folder to locate your manuscript file.

Please include the following items when submitting your revised manuscript:

* A letter that responds to each point raised by the editor and reviewer(s). You should upload this letter as a separate file labeled 'Response to Reviewers'. This file does not need to include responses to formatting updates and technical items listed in the 'Journal Requirements' section below.

* A marked-up copy of your manuscript that highlights changes made to the original version. You should upload this as a separate file labeled 'Revised Manuscript with Track Changes'.

* An unmarked version of your revised paper without tracked changes. You should upload this as a separate file labeled 'Manuscript'.

If you would like to make changes to your financial disclosure, competing interests statement, or data availability statement, please make these updates within the submission form at the time of resubmission. Guidelines for resubmitting your figure files are available below the reviewer comments at the end of this letter.

As the corresponding author, your ORCID iD is verified in the submission system and will appear in the published article. PLOS supports the use of ORCID, and we encourage all coauthors to register for an ORCID iD and use it as well. Please encourage your coauthors to verify their ORCID iD within the submission system before final acceptance, as unverified ORCID iDs will not appear in the published article. Only the individual author can complete the verification step; PLOS staff cannot verify ORCID iDs on behalf of authors.

We look forward to receiving your revised manuscript.

Kind regards,

Jae Young Choi

Academic Editor

PLOS Genetics

Justin Fay

Section Editor

PLOS Genetics

Aimée Dudley

Editor-in-Chief

PLOS Genetics

Anne Goriely

Editor-in-Chief

PLOS Genetics

**Additional Editor Comments (if provided):**

Reviewer 2 has noted misplaced citations. Please double check the possible errors.

**Reviewers' comments:**

Reviewer's Responses to Questions

**Comments to the Authors:**

**Please note here if the review is uploaded as an attachment.**

Reviewer #2: The authors have thoroughly addressed my previous concerns and comments. I think these additional analyses further enhanced the depth of this manuscript.

However, there is a significant typesetting error that must be corrected prior to publication:

Many of the cited references are mismatched between the main text and the reference section. For instance, references 61–67 are cited regarding amphioxus mutation rates, but entries 61–67 in the reference list are completely unrelated. Please perform a comprehensive check of all references to ensure the in-text citations match the reference list correctly.

Reviewer #4: The authors have addressed all my comments and I have no further comment.

**********

**Have all data underlying the figures and results presented in the manuscript been provided?**

Large-scale datasets should be made available via a public repository as described in the *PLOS Genetics*  data availability policy, and numerical data that underlies graphs or summary statistics should be provided in spreadsheet form as supporting information.

Reviewer #2: Yes

Reviewer #4: None

**********

PLOS authors have the option to publish the peer review history of their article (what does this mean?). If published, this will include your full peer review and any attached files.

If you choose “no”, your identity will remain anonymous but your review may still be made public.

**Do you want your identity to be public for this peer review?**  For information about this choice, including consent withdrawal, please see our Privacy Policy.

Reviewer #2: No

Reviewer #4: No

[NOTE: If reviewer comments were submitted as an attachment file, they will be attached to this email and accessible via the submission site. Please log into your account, locate the manuscript record, and check for the action link "View Attachments". If this link does not appear, there are no attachment files.]

**Figure resubmission:**

**Reproducibility:**

To enhance the reproducibility of your results, we recommend that authors deposit laboratory protocols in protocols.io, where a protocol can be assigned its own identifier (DOI) such that it can be cited independently in the future. Additionally, PLOS ONE offers an option to publish peer-reviewed clinical study protocols. Read more information on sharing protocols at https://plos.org/protocols?utm_medium=editorial-email&utm_source=authorletters&utm_campaign=protocols

# Author comment: Author response to Decision Letter 2

**DOI:** 10.1371/journal.pgen.1012293.r004

18 Aug 2026

Attachment Submitted filename: BrasoVives_etal_2026_Response2Reviewers.pdf

# Editor report: Decision Letter 2

**DOI:** 10.1371/journal.pgen.1012293.r005

**Authors:** Justin Fay, Jae Young Choi

19 Aug 2026

Dear Dr Brasó-Vives,

We are pleased to inform you that your manuscript entitled "The highly heterozygous European amphioxus (*Branchiostoma lanceolatum* ) at the edge of panmixia" has been editorially accepted for publication in PLOS Genetics. Congratulations!

Before your submission can be formally accepted and sent to production you will need to complete our formatting changes, which you will receive in a follow up email. Please be aware that it may take several days for you to receive this email; during this time no action is required by you. Please note: the accept date on your published article will reflect the date of this provisional acceptance, but your manuscript will not be scheduled for publication until the required changes have been made.

Once your paper is formally accepted, an uncorrected proof of your manuscript will be published online ahead of the final version, unless you’ve already opted out via the online submission form. If, for any reason, you do not want an earlier version of your manuscript published online or are unsure if you have already indicated as such, please let the journal staff know immediately at plosgenetics@plos.org.

In the meantime, please log into Editorial Manager at https://www.editorialmanager.com/pgenetics/, click the "Update My Information" link at the top of the page, and update your user information to ensure an efficient production and billing process. Note that PLOS requires an ORCID iD for all corresponding authors. Therefore, please ensure that you have an ORCID iD and that it is validated in Editorial Manager. To do this, go to ‘Update my Information’ (in the upper left-hand corner of the main menu), and click on the Fetch/Validate link next to the ORCID field.  This will take you to the ORCID site and allow you to create a new iD or authenticate a pre-existing iD in Editorial Manager.

If you have a press-related query, or would like to know about making your underlying data available (as you will be aware, this is required for publication), please see the end of this email. If your institution or institutions have a press office, please notify them about your upcoming article at this point, to enable them to help maximise its impact. Inform journal staff as soon as possible if you are preparing a press release for your article and need a publication date.

Thank you again for supporting open-access publishing; we are looking forward to publishing your work in PLOS Genetics!

Yours sincerely,

Jae Young Choi

Academic Editor

PLOS Genetics

Justin Fay

Section Editor

PLOS Genetics

Aimée Dudley

Editor-in-Chief

PLOS Genetics

Anne Goriely

Editor-in-Chief

PLOS Genetics

www.plosgenetics.org

BlueSky: @plos.bsky.social

----------------------------------------------------

Comments from the reviewers (if applicable):

----------------------------------------------------

**Data Deposition**

If you have submitted a Research Article or Front Matter that has associated data that are not suitable for deposition in a subject-specific public repository (such as GenBank or ArrayExpress), one way to make that data available is to deposit it in the Dryad Digital Repository. As you may recall, we ask all authors to agree to make data available; this is one way to achieve that. A full list of recommended repositories can be found on our website.

The following link will take you to the Dryad record for your article, so you won't have to re‐enter its bibliographic information, and can upload your files directly:

http://datadryad.org/submit?journalID=pgenetics&manu=PGENETICS-D-26-00218R2

More information about depositing data in Dryad is available at http://www.datadryad.org/depositing. If you experience any difficulties in submitting your data, please contact help@datadryad.org for support.

Additionally, please be aware that our data availability policy requires that all numerical data underlying display items are included with the submission, and you will need to provide this before we can formally accept your manuscript, if not already present.

----------------------------------------------------

**Press Queries**

If you or your institution will be preparing press materials for this manuscript, or if you need to know your paper's publication date for media purposes, please inform the journal staff as soon as possible so that your submission can be scheduled accordingly. Your manuscript will remain under a strict press embargo until the publication date and time. This means an early version of your manuscript will not be published ahead of your final version. PLOS Genetics may also choose to issue a press release for your article. If there's anything the journal should know or you'd like more information, please get in touch via plosgenetics@plos.org.

# Editor report: Acceptance letter

**DOI:** 10.1371/journal.pgen.1012293.r006

**Authors:** Justin Fay, Jae Young Choi

PGENETICS-D-26-00218R2

The highly heterozygous European amphioxus (*Branchiostoma lanceolatum* ) at the edge of panmixia

Dear Dr Brasó-Vives,

We are pleased to inform you that your manuscript entitled "The highly heterozygous European amphioxus (*Branchiostoma lanceolatum* ) at the edge of panmixia" has been formally accepted for publication in PLOS Genetics! Your manuscript is now with our production department and you will be notified of the publication date in due course.

The corresponding author will soon be receiving a typeset proof for review, to ensure errors have not been introduced during production. Please review the PDF proof of your manuscript carefully, as this is the last chance to correct any errors. Please note that major changes, or those which affect the scientific understanding of the work, will likely cause delays to the publication date of your manuscript.

Soon after your final files are uploaded, unless you have opted out or your manuscript is a front-matter piece, the early version of your manuscript will be published online. The date of the early version will be your article's publication date. The final article will be published to the same URL, and all versions of the paper will be accessible to readers.

For Research Articles, you will receive an invoice from PLOS for your publication fee after your manuscript has reached the completed accept phase. If you receive an email requesting payment before acceptance or for any other service, this may be a phishing scheme. Learn how to identify phishing emails and protect your accounts at https://explore.plos.org/phishing.

Thank you again for supporting PLOS Genetics and open-access publishing. We are looking forward to publishing your work!

With kind regards,

Janani Seenivasan

PLOS Genetics

On behalf of:

The PLOS Genetics Team

Carlyle House, Carlyle Road, Cambridge CB4 3DN | United Kingdom

plosgenetics@plos.org | +44 (0) 1223-442823

plosgenetics.org | Twitter: @PLOSGenetics
